# CHALLENGES AND STRATEGIES FOR HOME PAID CAREGIVERS: ADVANCING CARE FOR OLDER ADULTS IN URBAN CHINA

**DOI:** 10.1093/geroni/igae098.4333

**Published:** 2024-12-31

**Authors:** Zixuan Wu, Qingwei Wang

**Affiliations:** Johns Hopkins University, Baltimore, Maryland, United States; Xi’an Jiaotong-Liverpool University, Suzhou, Jiangsu, China

## Abstract

Older adults living in community settings often depend on family caregivers, especially those suffering from chronic illnesses. To mitigate caregiving burdens and secure professional nursing support, many families in China’s urban centers opt for high-end paid caregivers who offer integrated medical-care services. This study aims to explore the challenges faced by paid caregivers in providing care to older adults in Shanghai and to develop a framework for advancing caregiving for China’s aging population. Using a qualitative research design, this study draws on in-depth interviews with 10 paid caregivers from Yi Jia, a local aging care company that offers tailored nursing services. The interview data are supplemented with ethnographic observations and video recordings during home visits. Preliminary thematic analysis revealed three major themes: 1) Home-based care poses logistical challenges, including high technological demands that exceed the capabilities of caregivers from less developed regions and inadequate living conditions that compromise privacy and safety; 2) Paid caregivers face emotional burdens from the high demands of their work and must also cope with homesickness and limited social support due to their migration and separation from their families; 3) Cultural compatibility between caregivers, older adults, and families influenced by regional differences would lead to failure in accommodating local living habits and result in mismatches in health communication. Policymakers should regulate the paid caregiving market to ensure that caregivers are proficient in professional skills, emotional resilience, and cultural adaptability. Continuous collaboration between organizational supervisors, families, and caregivers is crucial to delivering comprehensive care.

